# Unusual clinical outcome of primary Hyperoxaluria type 1 in Tunisian patients carrying 33_34InsC mutation

**DOI:** 10.1186/s12882-017-0612-8

**Published:** 2017-06-15

**Authors:** Ibtihel Benhaj Mbarek, Saoussen Mdimeg, Amira Moussa, Dorsaf Zellama, Hayat Kaarout, Jaouida Abdelmoula, Abdellatif Achour, Saoussen Abroug, Asma Omezzine, Ali Bouslama

**Affiliations:** 1 Biochemistry Department, LR12 SP11, Sahloul University Hospital, 4054 Sousse, Tunisia; 2 Nephrology Department, LR12 SP11, Sahloul University Hospital, 4054 Sousse, Tunisia; 3 Internal Medicine A Department, Charles Nicolle University Hospital, Tunis, Tunisia; 4 Biochemistry Department, Charles Nicolle University Hospital, Tunis, Tunisia; 5 Pediatric Department, LR12 SP11, Sahloul University Hospital, 4054 Sousse, Tunisia; 6 Faculty of Pharmacy, University of Monastir, Monastir, Tunisia

**Keywords:** Primary hyperoxaluria type 1, Tunisian population, Urolithiasis, 33-34InsC mutation

## Abstract

**Background:**

Primary hyperoxaluria type 1 (PH1), is a rare and heterogeneous disease and one of major causes of renal insufficiency in Tunisia, caused by mutations in the *AGXT* gene. 33-34InsC mutation, was mainly described in children with a severe clinical feature leading to early death, but it was uncommonly reported in adult patients.

**Methods:**

Common mutations in *AGXT* were tested using PCR/RFLP technique in 111 patients (68 adult, 43 children) with suspected PH1.

**Results:**

We described 16 cases (eight adult and eight children) with a 33-34InsC mutation with a median age of 24 years [6 months - 73 years]. All children were in end stage renal disease (ESRD) at the median age of 3 years due to lithiasis and/or nephrocalcinosis. Unfortunately, 75% of them died with a median age of 2.5 years. For the majority of adults only spontaneous elimination of urolithiasis were noted, 37.5% preserved until now a normal renal function and 62.5% of them reached ESRD at the median age of 55.8 ± 12.31 years old.

**Conclusion:**

In this study 33-34InsC mutation gives a controversial clinical effect in children and adults. The implication of other genetic and/or environmental factors can play a crucial role in determining the ultimate phenotype.

## Background

Primary hyperoxaluria type 1 (PH1) is the common and severe form of hyperoxaluria. This rare autosomal recessive inborn disease is a result of glyoxylate metabolism defect, caused by an absence, deficiency or mislocalization of the liver-specific peroxisomal enzyme alanine:glyoxylate aminotransferase (AGXT). The kidney is the first organ affected by the massive rise in urinary oxalate through the genesis of recurrent stones and / or progressive nephrocalcinosis to finish in an early end-stage renal disease (ESRD) [1]. The increased plasma oxalate levels along the disease progress, and calcium oxalate deposition in various tissues leads to the systemic oxalosis, engendering serious complications as well as a fatal outcomes [2]. A combined liver-kidney transplantation is the unique cure of this defect. Isolated kidney transplantation leads ultimately to the recurrence of the disease [3].

PH1 has heterogeneous appearances and can affect patients whatever their age. The presentation varies from infantile nephrocalcinosis and failure to thrive, as a result of renal impairment, to a recurrent or only occasional urolithiasis [4] usually one half of patients experience ESRD at the time of diagnosis and 80% develop ESRD by the age of 3 years. Recurrent urolithiasis and progressive renal failure are mainly described in childhood or adolescence onset of PH1. However, the diagnosis of patients affected during adulthood is often altered, over 50% of them reach ESRD at the time of diagnosis, and are characterized by occasional stone passage [5, 6].

More than 178 pathological mutations in *AGXT* gene have been documented [7]. The 33-34InsC mutation is classified as severe by the fact that this insertion leads to a truncated protein without catalytic activity [8]. Clinically, 33-34InsC is known to be responsible for severe presentation and early death in childhood [9], although, it has also been described more rarely in adult patients.

In this report, we describe a controversial effect of the 33_34InsC mutation in Tunisian patients. We reported a pediatric cases with severe clinical forms and adults patients with a mild and unusual clinical course of PH1 toward ESRD.

## Methods

### Subjects

In this retrospective study we considered patients addressed to our department, between 2005 and 2012, for PH1 diagnosis. 43 children and 68 adult patients have been suspected for PH1 with different stage of chronic renal failure (CRF). The median age of diagnosed patients was 18 ± 16.74 years old with sex-ratio of 1.4. We consider in our study a highly selected cohort of patients and not a population-based sample, addressed by different pediatric nephrology and nephrology departments of Tunisia. Based on the rapid evolution of ESRD (especially for children), clinical and biochemical findings, and systemic oxalosis, PH1 is the most suspected diagnosis among our patients. In fact, all index cases were already with chronic renal failure (CRF) when diagnosed and required renal replacement therapy.

Urolithiasis and/or nephrocalcinosis were present in all cases. Elevated urinary oxalate or oxalate/creatinine ratio was noted in most of cases. Diagnosis was supported in some cases by renal biopsy and stone analysis. No liver biopsy was carried out on our patients because diagnosis was confirmed by genetic testing.

In order to decrease oxalate super saturation, most cases undertake systematically hyperhydration and to decrease oxalate synthesis, pyridoxine (vitamin B6) supplementation was indicated, but no Vit B6 responsiveness was tested.

### Molecular approach

After obtaining patients’ consent, genomic DNA was isolated from peripheral blood leucocytes, as described previously [10].

c.33_34InsC (p.Lys12GlnfsX156) was analyzed by amplification of genomic DNA and restriction enzyme digestion using previously documented [9] primers and conditions.

PCR detecting 74pb duplication in exon 1, was used to distinguish the major (Ma) and minor (Mi) alleles, and PCR/restriction enzyme test using *MwoI* was used to genotype for c.33_34InsC [9].

## Results

In this study, a molecular genetic diagnosis was performed in 111 index cases with suspected PH1. We reported 16 cases (16/118, 13.5%) identified with 33-34InsC mutation, nine of them (four children and five adult) were index cases and the seven others (four children and three adult) were diagnosed during familial investigations. The median age of patients was 24 years (range 0.5–73 years) with a sex-ratio of 0.6 (ten female and six male) and they were originated from central and south of Tunisia. Only one patient was in heterozygote state (I244T/ 33-34InsC), with Mi/Ma alleles. All the rest were homozygotes for 33-34InsC mutation and have the major alleles (Ma/Ma).

Clinically, PH1 has a variable presentation in our cohort and patients presented several symptoms at the time of diagnosis (Table 1). The detection of PH1 was dictated by unspecific symptoms of nephrolithiasis and renal insufficiency (RI). In fact, some signs corresponded mainly to manifestations of CRF, but other signs were not specific such as anemia, diarrhea, vomiting and general alteration.

**Table 1:** Phenotypic characteristics of 33-34insC mutation carriers

Family	N° patient	Circumstance of recruitment	Consanguinity	Circumstance of the diseases discovery	Renal echography	Oxaluria mmol/24H	Oxaluria/creatininuria mmol/mmol	Extra- renal alteration	Evolution (age)
*Pediatric cases*									
* Median Age of First symptoms*: *2.5 years (range 6 months to 4 years)*									
* Median Age at diagnosis*: *3 years (range 6 months to 12 years)*									
* F1*	1*	IC	3°	Anemia/RI	nephrocal	0.4	0.67	(−)	D (7 months)
* F2*	2	IC	1°	RI	lith + nephrocal	0.16	0,43	DG	D (4 yrs)
* F3*	3	IC	2°	RI	lith	0.57	0.026	OC	HD (7 yrs)
	4 (sister)	FS	2°	RI	lith	ND	ND	(−)	D (2 yrs)
	5 (sister)	FS	2°	RI	lith	ND	ND	(−)	D (3 yrs)
	6 (sister)	FS	2°	RI	lith	ND	ND	(−)	D (1 yrs)
* F4*	7	IC	2°	CN/AP	lith	0.73	ND	(−)	HD (5 yrs)
*Adult cases*									
* Median Age of First symptoms*: *46.5 years (range 7 to 70 years)*									
* Median Age at diagnosis*: *47.75 years (range 36 to 73 years)*									
* F5*	8	IC	0°	CN/RI	lith	ND	ND	HT	HD (61 yrs)
* F6*	9	IC	1°	CN/RI	lith	ND	ND	OC	HD (49 yrs)
	10 (father)	FS	1°	lith	lith	ND	ND	(−)	HD (73 yrs)
* F7*	11	IC	1°	CN/RI	lith	0.73	ND	(−)	HD (48 yrs)
	12 (niece)	FS	1°	lith	lith	ND	ND	(−)	D (12 yrs)
* F8*	13	IC	2°	CN	lith	2.55	ND	(−)	CRF (43 yrs)
	14 (brother)	FS	2°	lith	lith	Nl	ND	(−)	Nl
* F9*	15	IC	1°	CN	lith	Nl	ND	(−)	Nl
	16 (brother)	FS	1°	lith	lith	Nl	ND	(−)	Nl

*M* male; *F* female; *FS* family screening; *IC* index case; *RI* renal insufficient; *CN* colic nephritic ; *AP* abdominal pain; *lith* urolithiasis; *nephrocal* nephrocalcinosis; *ND* not done; *HT* hypertension; *DG* digestive; *OC* ocular; *HD* hemodialysis ; *CRF* chronic renal failure; *D* dead; *yrs* years. *The patient was heterozygous for 33 insC/ I244T mutations and has Ma/Mi haplotype. All other patients were homozygous 33 insC mutation and have Ma/Ma haplotype.

Oxalate/creatinine (mmol/mmol) ratio with relevant reference for age as reported by Belhaj et al. 2011 [27]: 0-6months: 0.36; 7-24 months: 0.17; 2-5 years: 0.10; >5 years: 0.081

### Pediatric cases

We report eight pediatric cases with a sex-ratio = 0.14 (seven girls and one boy), four of them were index cases and were addressed for RI. The four others were diagnosed during family investigation.

The median age of patients at presentation was 3 years (range 5 months - 12 years). Only one patient was in CRF stage, all the rest were already in ESRD at presentation.

Renal ultrasound exam detected nephrocalcinosis (one patient), a combined form of nephrocalcinosis and urolithiasis (one patient), while in the rest of patients we detected urolithiasis. Extra-renal complications were digestive and cardiac in two patients respectively.

Six of the eight patients had Ma/Ma alleles and carried 33_34insC in homozygote state. The patient F1–1, of a 5 month old month boy, has a Ma/Mi alleles and carried compound heterozygous 33_34insC/I244T mutations.

Unfortunately, 75% (6/8) of children died of their disease at the median age of 2.5 years, the rest are in ESRD.

**Fig. 1:**
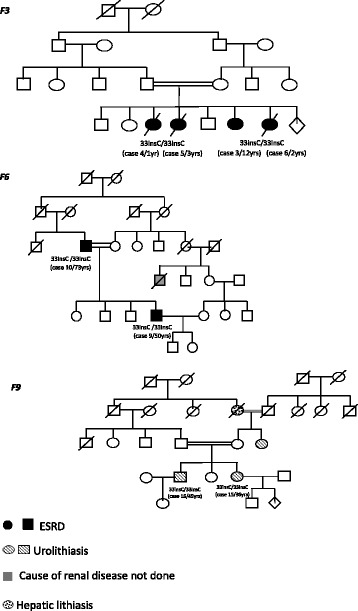
Pedigrees of families diagnosed for 33-34InsC mutation of PH1

The four cases discovered during the family investigations were all girls. Three of them belongs to the family F3 (Fig. 1), they suffered a severe form of urolithiasis with a rapid evolution to ESRD and death at a median age of 2 years [range 1–3 years]. The fourth case, (case 12), belong to the family F7 and was the niece of the adult patient (case 11). She had a history of recurrent urolithiasis since the age of 4 years, reached ESRD at the age of 8 and died of her disease at the age of 12 years.

### The adult cases

The eight adult cases were diagnosed in adulthood in the median age of 47 years (range 36 to 73 years) with a sex-ratio of 1.6 (three women and five men). Further, the median age of onset of ESRD was 55.8 years (range 43 to 73 years). Renal ultrasound exam detected urolithiasis in all patients. The main component of calculi, identified in patients 11 and 15, was whewellite (98%).

The history of urolithiasis was present in all the eight patients with detected 33-34insC and was the main cause of diagnosis in two of them. Two patients (cases 13 and 15) had their first renal colic in childhood (9 and 7 years, respectively). 3/8 patients had spontaneous elimination of calculi; while 5/8 of them suffered from recurrent nephrolithiasis, eliminated using extracorporeal shock wave lithotripsy.

Since the diagnosis 5/8 (62.5%) of our patients reached the ESRD, whereas 3/8 (37.5%) preserved until now a normal renal function with a median age of 40.3 years (range 36 to 45 years).

The three cases revealed during genetic family investigation presented a different feature of disease. The first was a man of 73 years-old, (case 10, Family F6) suffering CRF and having a history of spontaneous elimination of lithiasis (Fig. 1). Interestingly he preserved until 70 years-old a normal renal function. At his last following up the patient reached the ESRD. The second (case 14), a 40 year-old man, was the brother of case 13 and had a history of spontaneous elimination of urolithiasis with a preserved normal renal function. The third (case 16), was the brother of case 15 (48-year-old) (Fig. 1); First symptoms, as urolithiasis, appeared at the age of 45, eliminated by chirurgical intervention in 2013. In the last follow up (March 2016), the patient preserved a normal renal function and a normal value of oxaluria.

## Discussion

PH1, is a very rare inherited disease with a high prevalence in Mediterranean countries and Arabic nations due to genetic make-up and a higher rate of consanguineous marriages [11]. Tunisia is one of the countries with the highest frequencies of PH1 worldwide [12, 13]. Indeed, PH1 is responsible for more than 13% and 10% of ESRD in children in Tunisia and Kuwait versus 0.3% and 0.7% in, Europe and North America, respectively [12, 14].

Molecular genetic testing is the efficient and non-invasive approach, approved to confirm the definitive diagnosis of PH1. We identified the 33_34 InsC mutation in 13.5% (16/118) of our patients and in our knowledge, it is the highest frequency of patients carrying this mutation and having the same ethnicity reported until now. In particular interest, the 33_34InsC mutation was mainly described in pediatric cases (12%) and was very rarely reported in late onset particularly in the homozygote’s states [1, 15–17]. Compared to the others reports, we noted that the 33_34InsC mutation frequency in the Tunisian cohort is on par with that reported by Coulter-Mackie’s et al. (13.5%) [9], however the age of patients was mainly different. In fact the majority of patients in Coulter-Mackie’s cohort, were pediatric cases and only one adult women (54 years) was reported. In this report, we described 33_34InsC mutation in 17% (8/47) of pediatric cases and interestingly in 11.26% (8/71) of our adults. Otherwise, our results were slightly higher than those reported by Rumsby’s et al. (12%) [15], we have some similitudes for pediatric cases, but a difference in the number and the age of onset of the disease was mainly noted for adults. In fact, we reported more patients over 30 years- old.

This micro-insertion was considered as the most common PH1 mutation on the major allele (31%) of *AGXT* [9]. It has been reported that the severity of this micro-insertion, especially in homozygous state, is due to the production of no immune-reactive mature protein with no catalytic activity [8]. 33_34InsC mutation, was described firstly in the Italian population (of Roman origin) [18], then in other populations [19]. Given to her strategic geographical position and the historical invasive flows that took place in North Africa, Tunisia is considered as a crossroads of civilizations [20]. We hypothesize that 33_34InsC variation, is associated with a specific population or ethnic group and might have been introduced in Tunisia, as many other variations [21], during the Roman settlement up to the fifth century AC. Interestingly, 75% of our patients are originated from the cities (Kasserine, Mahdia, and Sfax), known for a long Roman colonization.

In our observation, the clinical manifestations were entirely different between children and adults patients and we noted the absence of the adolescent form. Interestingly, the pediatric patients suffered from a very severe clinical form caracterised by a renal symptoms ranged from urolithiasis, nephrocalcinosis or even a combined form, associated to rapid evolution to ESRD. Systemic oxalosis as ocular and digestive damaging were also present in two patients. Unfortunately, 75% of children carrying this mutation died with a median age of 2.5 years. Contrary to this instance, we noted the absence of the expected severity attributed to 33_34InsC mutation in adults’. Indeed, all of our adult patients have a slow development of the clinical symptoms of the PH1 characterised by urolithiasis. Owing to the lack of a strict medical follow-up and the delay of the PH1 diagnosis, five adults reached ESRD at the median age of 54 ± 8.7 years-old. Curiously, 37.5% (3/8) of our adult patients preserved a normal renal function until the age of 40 without B6 treatment. Only two of our patients, cases 13 and 15, have initial symptoms as recurrent lithiasis from the age of 9 and 7 years respectively. The patient 15 had preserved a normal renal function at last follow-up; however case 13 developed CRF at 43 years-old.

Strangely enough, a marked intra-familial clinical heterogenicity was noted, although the patients harbor the same AGXT mutations. Some are present with early and severe clinical manifestations, whereas others may be asymptomatic for long time. In fact, the case 11, was asymptomatic for many years and even with a near-normal urinary oxalate excretion, has first symptoms’ disease in the age of 48. However, her niece (case 12) died at the age of 12 years, after a history of urolithiasis and ESRD. For note, we have detected an uncommon case with pseudo-dominant inheritance in a consanguineous family (F6). With the same mutation and the same genotype (33-34insC/33-34insC), the clinical progression was different between patient and his father. In fact, at presentation, the case 9 started the CRF at the age of 50; however, his father preserved until the age of 70 a normal renal function. We have reported previously a cases of pseudo-dominant inheritance in families with I244T mutation [22], we suggested that consanguinity, strongly present in our population study (≈89%), has a serious effect in this great molecular heterogeneity of PH1.

Interestingly, this variation in the age of diagnosis and the reality of a moderately severe outcome for adult patients with no functional AGXT, supports the idea that other enzymes may be involved in oxalate synthesis, or that the metabolism of glyoxylate can be modulated by interactions with modifier genes and/or with environmental factors. These hypotheses have been occasionally mentioned [15], but we suppose that families described in this report could hold the key to further discoveries in PH.

Moreover, to maintain the renal function and avoid complications [23], several hypotheses were proposed to explain the effect of the diet as conservative measure to limit the quantity of oxalate precursors in the patient’s diet [24]. It was reported that oxalate-rich food restriction intakes is recommended for PH patients [25] because of their lower intestinal oxalate absorption, compared to normal subjects [24]. More than, it was appeared that the incidence of calcium lithiasis can be reduced by fiber intake that leads to a change in bowel transit and diminishes the absorption of both calcium and oxalate. For that reason, fiber intake is recommended for patients with recurrent lithiasis [23, 26]. Nevertheless, there is not enough scientific evidence to corroborate the benefits of this measure.

Looking for the diet of our adult cases having the alteration of the CRF stage under the age of 40, raises the question of the influence of the diet in this instance. Interestingly, the majority of adults are originated from West-central of Tunisia, the diet costumers in those regions are based on a rich fiber intake in their food as a whole wheat bread, spices, apples, barley; in addition to nutriment rich in vit B12 as fatty fish, lamb, offal, cereal, …

The limitation of our study was the shortage of information about the metabolic profile in adults. Unfortunately, the majority of them were addressed by different Tunisian nephrology departments for genetic analysis of PH1, but the biological measurement were unaffordable, at recruitment, because the majority of them reached ESRD. That restriction of data, make impossible the effective comparison of the metabolic profile between adults and children, in order to get more information on the causes of the variable clinical course.

## Conclusion

The 33_34InsC mutation causing primary hyperoxaluria type 1 is a severe mutation that seems to provide a high morbidity and ruthless infantile PH1. We report that 33_34insC can be present in adults’ forms with a mild clinical features, suggesting the implication of other factors in this slowly progression.

## Abbreviations

CRF: Chronic renal failure
ESRD: End stage renal disease
Ma: Major
Mi: Minor
PH1: Primary hyperoxaluria type 1
RI: Renal insufficiency
